# Influence of zinc levels and Nrf2 expression in the clinical and pathological changes in patients with diabetic nephropathy

**DOI:** 10.1038/s41387-022-00212-4

**Published:** 2022-08-06

**Authors:** Ping Nie, Yan Lou, Xue Bai, Yuexin Zhu, Qiaoyan Guo, Ping Luo, Weiguang Zhang, Bing Li

**Affiliations:** 1 Department of Nephropathy, The Second Hospital of Jilin University, Changchun, Jilin Province China; 2 Department of Nephrology, Chinese PLA General Hospital, Chinese PLA Institute of Nephrology, Beijing, China

**Keywords:** Kidney diseases, Nutrition disorders

## Abstract

**Objective:**

We investigated the correlation between zinc levels and Nrf2 expression and potential effects on the clinicopathology of patients with diabetic nephropathy (DN).

**Methods:**

We selected 30 patients with DN, diagnosed via renal biopsy at our hospital from March 2018 to November 2019, and enrolled 30 healthy individuals from a medical examination center as the control group. Patients with DN were divided into normal-zinc and low-zinc groups. We detected the levels of zinc, copper, and Nrf2 mRNA in their serum, and collected the clinical and pathological data of DN patients.

**Results:**

Serum zinc level and Nrf2 mRNA expression were significantly decreased in patients with DN compared to those of healthy people (*P* < 0.05). Of the 30 patients, 16 had low zinc (53.3%) and 14 had normal zinc levels (46.7%). There was no significant difference in the blood Nrf2 mRNA expression between the two groups (*P* > 0.05). However, the expression of Nrf2 in the kidney tissue of the low-zinc group was significantly lower compared to the normal-zinc group (*P* < 0.05). Diastolic blood pressure and copper levels were significantly higher in the low-zinc group (*P* < 0.05). In contrast, body mass index, red blood cell count, Hb level, and the ratio of zinc to copper were significantly lower in the low-zinc group (*P* < 0.05). The pathological classifications of the low-zinc group were more severe (*P* < 0.05).

**Conclusion:**

Patients with DN were more likely to have zinc deficiency and lower expression of Nrf2. Additionally, DN patients with zinc deficiency were prone to have more severe clinical and pathological manifestations.

## Introduction

Diabetic patients often present with zinc deficiency [1], which is primarily caused by the loss of zinc via urine and decreased zinc absorption in the gastrointestinal tract [2, 3]. Zinc, as an essential microelement, is critical for the normal structure, secretion, and function of insulin, and plays a role in protecting islet β cells from oxidative damage [4, 5]. Zinc is also an antioxidant and can directly protect cell membrane lipids and proteins and mercaptan-dependent macromolecules (tubulin and enzyme) from oxidative damage [6]. It can also induce other antioxidants, such as nuclear factor erythroid 2-related factor 2 (Nrf2).

Nrf2 is not only a master regulator of cellular antioxidant activity [7, 8], but it is also involved in the process of anti-apoptosis, and epithelial–mesenchymal transition (EMT) [9]. EMT, renal tubular atrophy, and dysfunction may cause loss of a considerable amount of nutrients, electrolyte imbalance, acid–base imbalance, and renal interstitial fibrosis, which are important factors for the progression of DN [10]. Our previous cell and animal experiments confirmed that zinc supplementation could activate Nrf2 and up-regulate its downstream antioxidant factors, thus reducing oxidative damage to renal tubular epithelial cells and kidney tissues [11].

At present, there are few studies on the role of zinc in patients with DN diagnosed via renal biopsy, especially on the relationship between zinc and Nrf2 expression in patients with DN. In this study, we sought to determine whether zinc deficiency affected Nrf2 expression in patients with DN and to ascertain the clinical and pathological features of DN patients with zinc deficiency. Therefore, we compared the plasma levels of zinc and Nrf2 between 30 patients with DN diagnosed via renal biopsy and healthy people. We also analyzed the expression of Nrf2 in the kidneys of DN patients with zinc deficiency to clarify the relationship between zinc and Nrf2 in these patients.

## Materials and methods

### Patients

We collected data from patients diagnosed with DN via renal biopsy at the Second Hospital of Jilin University from March 2018 to November 2019. All patients were diagnosed in accordance with the classification standard of diabetes developed by the American Diabetes Association in 2018 [12]. Patients with infection-related diseases, malignant tumors, severe hypertension, and severe liver dysfunction were excluded. The control group comprised healthy individuals from the physical examination center of our hospital, whose blood pressure, blood glucose, and blood lipids were in the normal range. All procedures involving human participants were in accordance with the ethical standards of the institutional and/or national research committee. Furthermore, all procedures were conducted according to the 1964 Helsinki Declaration and its later amendments or comparable ethical standards. Institutional Review Board approval (2019095) was provided prior to study onset. Informed consent was obtained from all participants included in the study.

### Blood analysis and biochemistry analysis

We took the patient’s blood sample on the morning of the day of the renal biopsy. Both the patient and control groups were assessed for plasma trace elements, including zinc and copper. The test was performed by Adicon Clinical Laboratories (Hangzhou, China) through atomic absorption spectrometry. The reference value of serum zinc was 76.5–170 μmol/L and the reference value of serum copper was 11.8–39.3 μmol/L.

We retrospectively analyzed the sex, age, course of the disease, physical examination data, and laboratory data. Data from healthy people were obtained from the results of their physical examination. Data of patients with DN were the latest results before the renal biopsy. Physical examination included measurement of body mass index (BMI), systolic blood pressure (SBP), and diastolic blood pressure (DBP). Laboratory parameters included hemoglobin (Hb), red blood cell (RBC), albumin (Alb), serum creatinine (Scr), blood urea nitrogen, uric acid, triglycerides (TG), total cholesterol (TC), and glycated hemoglobin (HbA1c) levels, and 24 h urinary protein quantification. The estimated glomerular filtration rate (eGFR) was expressed as milliliter/(min/1.73 m^2^) and was calculated by the simplified MDRD formula: eGFR = 186×(Scr/88.402)^−1.154^ × age^−0.203^ × (0.742 if female).

### Kidney histopathology

The biopsy specimens of patients with DN were processed and stained with hematoxylin and eosin, periodic acid-Schiff, periodic acid-Schiff-methenamine, and Masson’s trichrome solution. We also examined the degree of thickening of basement membranes and the range of foot process fusion using a JEM-1400 Plus electron microscope. The pathological diagnosis and classification were based on the 2010 DN pathological diagnosis standard [13]. The interstitial lesions comprised interstitial fibrosis and tubular atrophy, and renal interstitial inflammation. Vascular lesions comprised atherosclerosis and arteriolar hyalinosis.

### Reverse transcription-quantitative polymerase chain reaction (RT-qPCR) analysis

We detected the mRNA level of Nrf2 in blood by RT-qPCR. Total RNA was extracted from blood stored at −80 °C with the TRIzol reagent (TIANDZ, Beijing, China). RNA was reverse transcribed to cDNA using the All-in-One RT MasterMix (ABP Biosciences, Rockville, MD, USA). Real-time PCR was performed using the SYBR Green qPCR Master Mix (ABP Biosciences) and the ABI7300 Real-Time qPCR system. All PCR experiments were performed in triplicate. Primer sequences were as follows: Nrf2: forward 5′-TGCATGATGCCCAATGTGA-3′, reverse 5′-CCAAGCGGCTTGAATGTTT-3′.

### Immunohistochemical (IHC) staining

We detected the expression of Nrf2, HO-1 in renal tissue by immunohistochemical (IHC) staining. Paraffin-embedded kidney tissue sections were incubated with the primary anti-Nrf2 antibody (1:100; Abcam, Cambridge, MA, USA) and anti- Heme Oxygenase 1 antibody (1:100; Abcam, Cambridge, MA, USA) overnight at 4 °C. The sections were washed with phosphate-buffered saline containing 0.1% Triton (PBS-T) and were incubated with a secondary goat anti-rabbit antibody (1:200; Bioss, Beijing, China) for 1 h at 25 °C. After washing with PBS-T, the sections were stained with diaminobenzidine (DAB).

### Statistical analysis

SPSS version 25.0 (Chicago, IL, USA) was used for data analysis. Continuous variables were presented as the mean ± standard error if they obeyed a normal distribution. Student’s *t*-test was used to compare the differences between the two groups. Median and quartile ranges were used to represent the continuous variables that did not obey a normal distribution. The Wilcoxon rank-sum test was used to compare the differences between the two groups. The Pearson correlation analysis was used to discuss the correlation between zinc and clinical indicators. Categorical variables were presented as percentages and were compared using the chi-square test. The data were graphed using GraphPad Prism 7.0 (GraphPad Software Inc., San Diego, CA, USA). A *P* < 0.05 was considered significant.

## Results

### Comparison of plasma zinc levels between patients with DN and healthy people

**Fig. 1:**
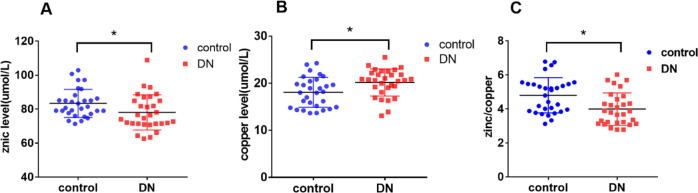
Scatter diagrams showing the plasma levels of zinc, copper, and zinc/copper. **A** Plasma zinc levels were significantly decreased in the DN group compared to those in the control group (*P < 0.05). **B** Plasma copper levels were significantly increased in the DN group compared to those in the control group (*P < 0.05). **C** The ratio of zinc and copper was significantly decreased in the DN group compared to that in the control group (*P < 0.05).

The levels of zinc and ratio of zinc to copper (zinc/copper) in the DN group were significantly lower than those in the control group, while the levels of copper were higher in the DN group than in the control group (*P* < 0.05) (Fig. 1). We then analyzed other indices of the two groups. The positive rates of TC and Scr urinary proteins were significantly higher in the DN group than in the control group, while the levels of Alb were significantly lower in the DN group than in the control group (*P* < 0.05, respectively).

### Comparison of Nrf2 expression between patients with DN and healthy people

**Fig. 2:**
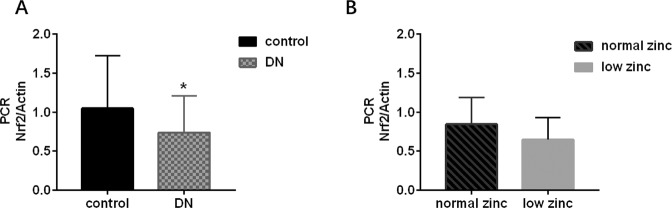
Effect of zinc on Nrf2 expression in the blood of patients with DN. **A** RT-qPCR was performed to evaluate mRNA expression of Nrf2 in the blood of patients with DN and healthy people. **B** The mRNA expression of Nrf2 in the blood of patients with DN in the normal-zinc and low-zinc groups. Data are presented as means ± SD, *P < 0.05 versus the control group.

Real-time PCR assessment of blood showed that Nrf2 mRNA expression significantly decreased in patients with DN compared to that in healthy people (Fig. 2A, *P* < 0.05).

### Relationship of zinc with Nrf2 expression in patients with DN

**Fig. 3:**
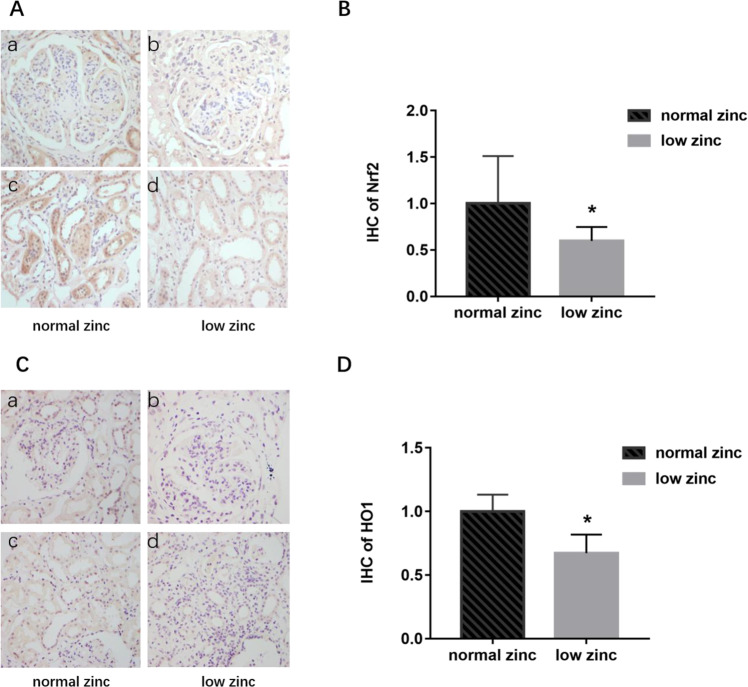
Effect of zinc on Nrf2 expression in the kidneys of patients with DN. IHC staining of Nrf2 and semiquantitative analysis were performed to evaluate the expression of Nrf2 and HO1 in the kidneys of patients with DN. **A** The expression of Nrf2 in the glomeruli and tubules (IHC staining *400). **B** The expression of Nrf2 in the renal tissue of the low-zinc group was significantly lower than that in the renal tissue of the normal-zinc group. *P < 0.05 versus the normal-zinc group. **C** The expression of HO1 in the glomeruli and tubules (IHC staining *400). **D** The expression of HO1 in the renal tissue of the low-zinc group was significantly lower than that in the renal tissue of the normal-zinc group. *P < 0.05 versus the normal-zinc group.

Of the 30 patients with DN, 16 were in the low-zinc group (53.3%), and 14 were in the normal-zinc group (46.7%). The expression of Nrf2 mRNA in the low-zinc group was slightly lower than that in the normal-zinc group, but there was no significant difference (Fig. 2B, *P* > 0.05). We also verified this result by ELISA (Fig. S1, *P* > 0.05). Additionally, IHC staining of Nrf2 and HO-1 in the kidney sections indicated that the expression of Nrf2 and HO-1was lower in the low-zinc group than in the normal-zinc group (Fig. 3, *P* < 0.05).

### Relationship of zinc with clinical and laboratory features of patients with DN

**Fig. 4:**
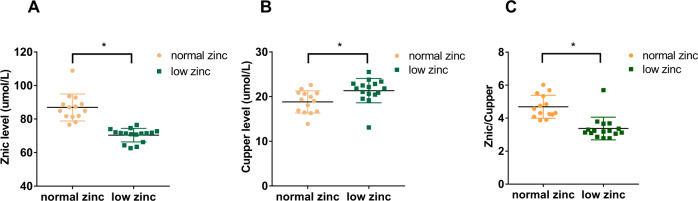
Scatter diagrams showing the plasma levels of zinc, copper, and zinc/copper of patients with DN. **A** Plasma zinc levels were significantly lower in the low-zinc group than in the normal-zinc group (*P < 0.05). **B** Plasma copper levels were significantly higher in the low-zinc group than in the normal-zinc group (*P < 0.05). **C** The ratio of zinc and copper in the low-zinc group was significantly lower than that in the normal-zinc group (*P < 0.05).

**Fig. 5:**
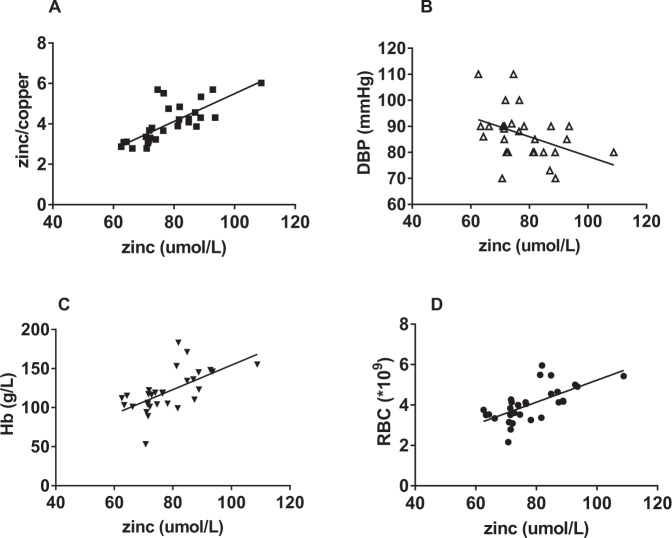
Correlation between zinc levels and clinical indicators of patients with DN. According to the Pearson correlation analysis, zinc was **A** positively correlated with zinc/copper (*r* = 0.75, *P* < 0.01); **B** negatively correlated with DBP (*r* = −0.409, *P* < 0.05); **C** positively correlated with Hb (*r* = 0.617, *P* < 0.01); and **D** positively correlated with RBC (*r* = 0.652, *P* < 0.01).

Further analyses were performed to determine the relationship of zinc with clinical and laboratory features. The levels of copper and DBP were significantly higher in the low-zinc group than in the normal-zinc group (*P* < 0.05, respectively). In contrast, the levels of zinc/copper, the proportion of men, BMI, Hb levels, and RBC count were significantly lower in the DN patients with low zinc levels than in the DN patients with normal zinc levels (*P* < 0.05, respectively; Table 1 and Fig. 4). According to the Pearson correlation analysis, zinc was positively correlated with RBC count, Hb levels, and zinc/copper levels (*r* = 0.652, 0.617, and 0.75, respectively; *P* < 0.01) and was negatively correlated with DBP (*r* = −0.409; *P* < 0.05) (Fig. 5).

**Table 1:** Clinical data of low-zinc and normal-zinc groups.

	Normal zinc (*n* = 14)	Low zinc (*n* = 16)	*P-*value
Male (*n*, %)	10 (71.4%)	4 (25.0%)	0.026*
Age	48.3 ± 13.8	45.3 ± 11.8	0.530
History of diabetes (years)	7.9 ± 6.4	10.3 ± 7.8	0.384
BMI	27.0 ± 4.1	23.5 ± 4.1	0.033*
SBP (mmHg)	135.0 (130.0,162.5)	140.0 (130.5,157.5)	0.785
DBP (mmHg)	83.1 ± 7.7	90.0 ± 10.2	0.045*
RBC (*10^9^)	4.6 ± 0.8	3.5 ± 0.5	<0.001*
Hb (g/L)	140.5 (116.8,153.5)	105.0 (101.0,116.8)	0.001*
Plt (*10^11^)	254.5 (209.1,292.5)	265.5 (194.1,309.2)	0.901
Alb (g/L)	34.9 (24.5,45.9)	27.6 (24.1,33.4)	0.146
TG (mmol/L)	1.8 (1.4,2.6)	1.9 (1.4,2.3)	0.835
TC (mmol/L)	5.7 ± 1.7	6.6 ± 1.8	0.154
HbA1c (mg/dl)	7.0 (6.4,8.2)	7.3 (5.9,8.1)	0.917
UA(μmol/L)	385.3 ± 67.1	340.3 ± 81.1	0.113
Scr (μmol/L)	111.4 ± 43.8	125.3 ± 50.6	0.432
BUN (mmol/L)	7.1 (6.0,11.3)	8.3 (6.7,11.2)	0.618
eGFR [mL/(min.1.73 m^2^)]	70.4 ± 30.6	57.6 ± 30.3	0.259
24-h urinary protein (g)	5.0 ± 3.9	6.2 ± 2.9	0.333

Sex of the two groups was compared by the chi-square test. Age, history of diabetes, BMI, DBP, RBC, TC, UA, Scr, eGFR, and 24-h urinary protein of the two groups were compared by *t*-test. SBP, the level of Hb, Plt, Alb, TC, HbA1c, BUN, copper, and zinc/copper of the two groups were compared by the Wilcoxon rank-sum test.

*BMI* body mass index, *SBP* systolic blood pressure, *DBP* diastolic blood pressure, *Hb* hemoglobin, *Alb* plasma albumin, *TC* total cholesterol, *TG* triglyceride, HbA1c glycated hemoglobin, *UA* uric acid, Scr serum creatinine, BUN blood urea nitrogen, eGFR estimated glomerular filtration rate.

*P < 0.05.

### Effect of zinc on pathological changes in the kidney

**Fig. 6:**
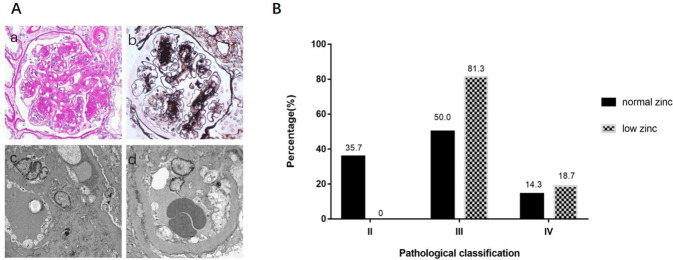
Pathological classifications of patients with DN. **A** Typical pathological change of DN (PAS staining*400, Masson staining *400, EM*6000). **B** In general, the pathological classification of the low-zinc group was more serious than that of the normal-zinc group.

The pathological classification of the low-zinc group was more serious than that of the normal-zinc group. In the normal-zinc group, there were five cases of type II (including two cases of IIa and three cases of IIb), seven cases of type III, and two cases of type IV. However, in the low-zinc group, there were no cases of type II, 13 cases of type III, and 3 cases of type IV (Fig. 6 and Table 2). Additionally, the incidence of K-W nodules and capillary microaneurysms was greater in the low-zinc group than that in the normal-zinc group (*P* < 0.05). There was no significant difference in mesangial dissolution, capsular hyaline drop, the ratio of glomerulosclerosis, interstitial lesions, and vascular lesions between the two groups (Table 2).

**Table 2:** Relationship of zinc with pathological characteristics.

	Normal zinc (*n* = 14)	Low zinc (*n* = 16)	*P value*
*Pathological classification*			0.032*
II [*n* (%)]	5 (35.7%)	0 (0%)	
III [*n* (%)]	7 (50.0%)	13 (81.2%)	
IV [*n* (%)]	2 (14.3%)	3 (18.7%)	
*Mesangial expansion with increased matrix*			0.003*
Mild [*n* (%)]	2 (14.3%)	0 (0%)	
Moderate [*n* (%)]	9 (64.3%)	4 (25.0%)	
Severe [*n* (%)]	3 (21.4%)	12 (75.0%)	
K–W nodules [*n* (%)]	9 (64.3%)	16 (100%)	0.014*
Capillary microaneurysms [*n* (%)]	5 (35.7%)	13 (81.3%)	0.024*
Mesangial dissolution [*n* (%)]	1 (7.1%)	3 (18.8%)	0.352
Capsular drop, hyaline [*n* (%)]	5 (35.7%)	4 (25.0%)	0.694
Glomerulosclerosis (%)	22.1 (9.8,33.3)	13.1 (3.8,42.7)	0.406
Interstitial lesions	3.3 (2.0,4.0)	3.3 (3.0,4.0)	0.715
Vascular lesions	3.0 (2.0,3.0)	2.0 (2.0,3.0)	0.586
Diffuse thickening of GBM	6 (42.9%)	10 (62.5%)	0.586
Foot process extensive fusion	5 (35.7%)	5 (31.3%)	1.000

K–W nodules Kimmelsteil–Wilson nodules.

*P < 0.05. By chi-square and Wilcoxon rank-sum test.

## Discussion

Zinc is an essential trace element in the human body. Most zinc ions are tightly bound to proteins in cells, and only a small amount exists in the free form [14]. Zinc participates in the formation of multiple enzymes, maintains homeostasis of the body, regulates gene expression, and mediates cell signal transduction [15].

People with diabetes are prone to zinc deficiency [1, 16]. In our study, we concluded that the plasma level of zinc in patients with DN was significantly lower than that in the control group. The imbalance of zinc homeostasis is associated with type 2 diabetes and insulin metabolic disorders [5]. Zinc transporter 8 (ZnT8) is essential for the structure and insulin secretion of pancreatic β cells, representing an attractive target for diabetes therapy [17]. We surmised that the plasma level of copper was significantly higher in patients with DN than in the control group. Plasma zinc/copper in patients with DN was significantly lower than that in the normal group. Thus, zinc/copper may be a superior indicator for human metabolism compared with zinc or copper status alone. Further, zinc/copper is positively associated with estimated glomerular filtration rate, and negatively associated with HbA1c levels [18].

Nrf2 is a master regulator of cellular antioxidant activity. Under normal conditions, Nrf2 is localized in the cytoplasm and is mainly bound to epoxy chloropropane Kelch sample-related protein-1 (KEAP1). When oxidative stress increases, Nrf2 dissociates from KEAP1 and enters the nucleus; this increases the expression of downstream antioxidant genes and resists oxidative damage [19]. Increasing evidence shows an effect of zinc on diabetic complications, seemingly through Nrf2. It is demonstrated that the Nrf2-ARE signal transduction pathway increases ZnT-1, ZnT-3, and ZnT-6 mRNA levels, and decreases ZnT-10 and ZIP-3 mRNA levels [20]. In this study, Nrf2 mRNA expression was found to be significantly decreased in the blood of patients with DN. The expression of Nrf2 mRNA in the low-zinc group was slightly lower than that in the normal-zinc group, but the difference was not significant; this may be attributed to the limited sample size. However, there was low Nrf2 and HO-1 expression in the kidneys of DN patients with low zinc levels, suggesting that zinc levels are related to the expression of Nrf2, and both low zinc levels and DN reduce the ability of antioxidant damage.

In this study, we found that women with DN are more likely to have a zinc deficiency. In a study, Luo et al. [21]. showed that women are at a higher risk of zinc metabolism disorders. McNair et al. [22]. showed that serum zinc levels were lower in women because their urine zinc excretion was higher than that in men. Moreover, it was reported that the level of zinc in obese people is reduced [23]. Our study showed that the BMI of patients with low zinc level-associated DN was relatively low, but through the Pearson correlation analysis, we found there was no significant correlation between BMI and zinc levels. Thus, larger sample size is required to validate this finding.

We found that DN patients with low zinc levels were more likely to have higher DBP. It has been reported that zinc plays a substantial role in the prevention of elevated blood pressure [24, 25] concluded that serum zinc levels are not associated with SBP, but individuals with lower levels of serum zinc have significantly higher DBP than others. We also found that DN patients with low zinc levels had significantly lower Hb levels and RBC counts than those with normal zinc levels. In recent years, clinical studies have suggested that zinc level-associated disorders are related to a variety of hematopoietic-related diseases [26, 27]. A variety of zinc finger transcription factors are necessary for protein expression during the maturation of terminal erythrocytes [28].

Additionally, zinc levels and copper/zinc correlated with the severity of the disease. Zinc and copper are antagonistic microelements because they compete for metallothionein, the same carrier protein, during intestinal absorption. The increase in copper absorption leads to competitive inhibition of the absorption of zinc in the intestine, leading to zinc absorption disorder in the intestine, thus increasing the ratio of copper to zinc. The results of our study also corroborated this finding. The zinc/copper ratio is considered an important index of prognosis and recurrence of diabetes [18].

We concluded that the pathological classifications, the extent of mesangial expansion, the incidence of K–W nodules, and capillary microaneurysms of the low-zinc group were more serious and evident than those of the normal-zinc group. In diabetes, nonenzymatic glycation reactions occur between the amino group of proteins, fatty acids, or nucleic acids and the aldehyde group of reducing sugars, resulting in an increase of advanced glycation end products (AGEs) [29, 30]. Mesangial cells can synthesize and secrete matrix components simultaneously with uptake and degradation of AGEs, resulting in an increase of mesangial matrix components. AGEs accumulate in the expanded mesangial matrix, thickened glomerular basement membranes, and nodular lesions [31]. It has been reported that zinc supplementation may be beneficial to AGE-induced endothelial cell injury, probably through enhancement of intracellular NO production and down-regulation of NF-κB activation [32]. Therefore, we suggest that zinc deficiency may also increase the mesangial matrix of DN by aggravating AGEs. Additionally, mesangial cells can produce extracellular matrix (ECM) proteins [33]. Hyperglycemia promotes the deposition of ECM and the inhibition of matrix metalloproteinase, thus leading to expansion of mesangial matrix and thickening of glomerular basement membrane [34]. It has also been reported that zinc deficiency can aggravate tubulointerstitial fibrosis in diabetes [35]. Nrf2 negatively regulates ECM [7]. The activation of Nrf2–ARE pathway help to resist HG-induced up-regulation of FN and ICAM-1 in GMCs and diabetic mice kidneys [36]. We found that Nrf2 expression was lower in DN patients with zinc deficiency, which resulted in the accumulation of ECM. Therefore, the pathological classifications and manifestations of DN patients with zinc deficiency were more serious than those of the normal-zinc group were. The sample size of our study is not particularly large because only a small number of DN patients underwent renal biopsy during the study period. In future work, we will continue to collect the data and samples from DN patients, and continue to explore the mechanism by which zinc and Nrf2 affect the progress of DN.

## Conclusion

This study provides current information on the relationship between clinicopathological features and plasma zinc levels in patients with DN diagnosed via renal biopsy in Northeast China. We also identified that patients with DN were more likely to have zinc deficiency. The expression of Nrf2 was lower in patients with DN, especially in DN patients with zinc deficiency. Additionally, DN patients with zinc deficiency were prone to more severe clinical and pathological manifestations.

## Supplementary information

FigureS1 legend

Figure s1

## Data Availability

The data used to support the findings of this study are available from the corresponding author upon request.
