# Supplementary material for: Influence of zinc levels and Nrf2 expression in the clinical and pathological changes in patients with diabetic nephropathy
Source: Nutr Diabetes. 2022 Aug 6;12:37. doi: 10.1038/s41387-022-00212-4 (PMC9357008; doi:10.1038/s41387-022-00212-4)
Supplement: Supplementary file 1 — FigureS1 legend [file 41387_2022_212_MOESM1_ESM.doc]

**Figure legends**

**Figure s1.** The level of Nrf2 in blood of patients with DN in the normal-zinc and low-zinc groups by ELISA. Data are presented as means ± SD, **P* < 0.05 versus normal-zinc group.
