# Supplementary figures and images for: Influence of zinc levels and Nrf2 expression in the clinical and pathological changes in patients with diabetic nephropathy
Source: Nutr Diabetes. 2022 Aug 6;12:37. doi: 10.1038/s41387-022-00212-4 (PMC9357008; doi:10.1038/s41387-022-00212-4)

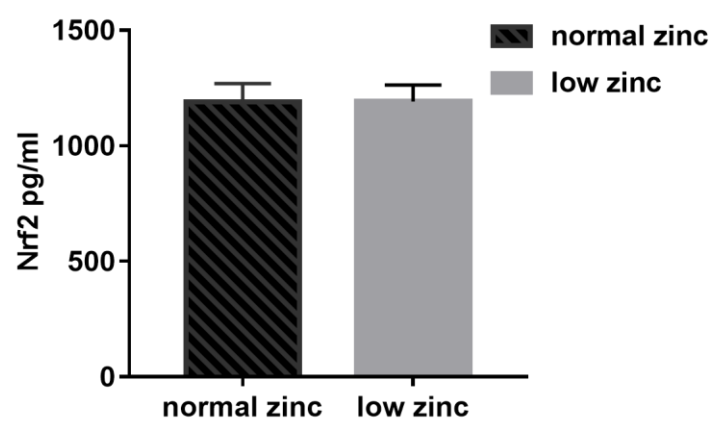

Figure S1

Supplement: Supplementary file 2 — Figure s1 [file 41387_2022_212_MOESM2_ESM.pdf]
